# Safety and tolerability of repeated sessions of deep transcranial magnetic stimulation in obesity

**DOI:** 10.1007/s12020-020-02496-x

**Published:** 2020-09-22

**Authors:** Anna Ferrulli, Stefano Massarini, Concetta Macrì, Livio Luzi

**Affiliations:** 1 Department of Biomedical Sciences for Health, University of Milan, Via Mangiagalli 31, 20133 Milan, Italy; 2 Present Address: Department of Endocrinology, Nutrition and Metabolic Diseases, IRCCS MultiMedica, Via Milanese 300, 20099 Sesto San Giovanni (MI), Italy

**Keywords:** Transcranial magnetic stimulation, Safety, Adverse events, Obesity

## Abstract

**Purpose:**

Repetitive Transcranial Magnetic Stimulation (rTMS) has been demonstrated to be effective in body weight control in individuals with obesity. Most clinical trials on rTMS provided a reassuring safety profile. In the present work, we present an extensive analysis on both severe and mild Adverse Events (AEs) in obese individuals treated with rTMS.

**Methods:**

We examined the intensity, duration, correlation with the treatment, up to 1 year after the end of rTMS treatment.

**Results:**

Descriptive analysis included a total of 63 subjects undergoing a 5-week deep rTMS experimental treatment for obesity (age 48.3 ± 10.4 years; BMI 36.3 ± 4.4 kg/m^2^): 31 patients were treated with high-frequency rTMS (HF), 13 with low-frequency rTMS (LF), and 19 were sham treated (Sham). Thirty-two subjects (50.8%) reported a total of 52 AEs, including mainly moderate (51.9%) events. The most frequently reported side effects were headaches of moderate intensity (40.4%) and local pain/discomfort (19.2%) and resulted significantly more frequent in HF group compared to other groups (*p* < 0.05). No significant differences among groups were found for the other reported AEs: drowsiness, insomnia, paresthesia, vasovagal reactions, hypertensive crisis. No AEs potentially related to the rTMS arised up to 1 year from the end of the treatment.

**Conclusions:**

This is the first comprehensive safety analysis in obese patients treated with rTMS. The analysis did not reveal any unexpected safety concerns. Only headaches and local pain/discomfort have been significantly more frequent in the HF group, confirming the good tolerability of rTMS even in the obese population potentially more susceptible to side effects of brain stimulation.

## Highlights

- A safety analysis in obese individuals treated with TMS did not reveal any unexpected safety concern up to 1 year after the end of the treatment.
- Only headache and local pain/discomfort have been significantly more frequent in the high-frequency TMS-treated group compared with low-frequency and sham-treated groups.
- TMS exhibits a good safety profile in the obese population potentially more susceptible to side effects of brain stimulation.

## Introduction

Transcranial magnetic stimulation (TMS) is a neurostimulation and neuromodulation technique, based on the principle of electromagnetic induction of an electric field, generated by a coil placed over the scalp [1]. In view of its magnitude and density, this field is able to depolarize neurons; furthermore, the application of repetitive TMS (rTMS) pulses could modulate cortical excitability, decreasing or increasing it, according to the parameters of stimulation: intensity of the stimulus, pulse frequency, duration of the stimulus train, time interval between trains. For example, when applied at a low frequency (≤1 Hz), TMS suppresses cortical excitability, while high-frequency TMS (≥5 Hz) enhances cortical excitability [2].

Neurostimulation outcomes could be affected also by the coil characteristics: the standard TMS with round and figure-of-eight coil has been shown to be effective to stimulate brain areas at the depth 2–2.5 cm from the scalp. However, the size of the magnetic field generated by this coil is not sufficient to reach the deeper cortical, subcortical, and limbic areas [3]. Even by using standard TMS coils with boosted outputs, a safe stimulation of much deeper brain sites would not be guaranteed, as the intensity required, using standard TMS coils, could lead to undesirable side-effects induced in the more superficial regions [4]. These limitations have led to the development of novel coil designs, as the H-coil, able to directly stimulate much larger and deeper brain regions by significant reduction of the decay rate [3]. Specifically, H-coil generates summation of the electric field in a specific brain region at a depth of 4–6 cm from the scalp, notwithstanding entailing higher and wider spread electrical field in superficial cortical regions [3]. Although this may suggest a higher susceptibility to side effects in patients treated with deep TMS than with standard TMS, no significant differences in safety and tolerability arose between the two neurostimulation technique, provided the guidelines are respected [5].

For these properties, rTMS, especially deep TMS, has been shown to have therapeutic benefits for several neuropsychiatric disorders [6], and has recently been proposed as a potential treatment in neuropsychiatric disorders associated with intracerebral dopamine deficiency, such as addiction disorders [7–9], control of food craving [10] and obesity [11, 12].

In the last two decades, the number of applications of conventional TMS has grown impressively, as well as the number of subjects who have undergone clinical trials aimed at exploring new medical therapeutic applications of TMS [1]. Given the high number of TMS applications and the heterogeneity of the stimulation protocols, a group of European experts has recently drawn up the guidelines on the therapeutic use of rTMS, establishing that there is a sufficient level of evidence to recommend the use of rTMS in several neurological, and psychiatric domains [13].

The majority of studies on clinical applications of TMS agree on its safety profile, which is supported by several meta-analyses [14–16]. Considering the expanding use of TMS, safety guidelines and recommendations of practice, have been revisited and updated in a consensus conference which took place in 2008 [1].

The most serious safety hazard of TMS is its potential to cause seizures. Seizures are induced by hypersynchronized discharges of groups of neurons in the gray matter, mainly due to an imbalance between inhibitory and excitatory synaptic activity, with prevalence of the latter [1]. Up to 2008, a total of 16 cases of seizures had been identified. Seven of these cases were included in the 1998 safety guidelines, and 9 of them were reported in the following years [1]. More recently, a survey specifically investigated the risk of seizures from TMS, and estimated that TMS, delivered within published guidelines, caused fewer than 1 seizure per 60,000 sessions [17]. Seizures appear to be more probable when safety guidelines are not observed. In fact, seizures were most likely to occur during the first few exposures to TMS, or when pulses are applied with high frequencies and short interval periods between trains of stimulation [17, 18].

Among uncommon severe AEs, hearing impairment and vasodepressor (neurocardiogenic) syncope have been also reported [1, 19]. Each TMS pulse produces a strong clicking noise, due to rapid mechanical deformation of the TMS stimulation coil, that could be responsible of transient changes in auditory threshold, mainly in subjects not using hearing protection [19]. TMS-associated syncope events are rare, but several cases have been reported in the literature [20–22]. Those episodes could likely be often related to anxiety and psychophysical discomfort during the procedure [1].

Effects on psychiatric behavior, immune system, autonomic function have less frequently occurred in association to rTMS. Local pain at the site of magnetic stimulation during the procedure, headache, neck pain, and discomfort represent the most common side effects occurring during a course of rTMS [19].

Most safety studies have investigated the side effects of TMS in individuals suffering from neurological and/or psychiatric disorders; a good tolerability of neurostimulation has also been observed in healthy volunteers [23]. A specific study on the rTMS side effects in obesity has not been previously conducted. To note that subjects with obesity exhibit an altered sensory detection and pain sensitivity [24]. Specifically, obesity was associated with several painful syndromes, including chronic pain, fibromyalgia, low back pain, neck pain, and migraine [25]. Several mechanisms appear to be involved in increased pain sensitivity in individuals with obesity, such as metabolic and inflammation mechanisms, genetic, environmental, behavioral, and socio-cultural factors [26]. Therefore, it is conceivable that obese subjects elicit an increased susceptibility to side effects of rTMS.

In a recent randomized clinical trial, we demonstrated the efficacy of 5 weeks of high-frequency deep TMS (HF-dTMS) treatment in reducing body weight up to 1 year in a population of individuals with obesity, with the modulation of the dopaminergic pathway and stimulation of physical activity as effectors mechanisms [11]. In this study, we present our safety and tolerability data, collected up to 1 year after the end of the treatment, in subjects with obesity undergoing deep rTMS.

## Materials and methods

### Study design

Data for this analysis were collected from January 2017 through January 2020 in the Endocrinology and Metabolic Diseases outpatient clinic for overweight/obesity treatment, at the IRCCS Policlinico San Donato (San Donato Milanese, Italy).

This study was conducted in accordance with the ethical standards of the Institutional Research Committee and with the 1964 Helsinki declaration and its later amendments; it received approval from the local institutional review board (Ethics Committee of San Raffaele Hospital, Milan, Italy). All participants provided written informed consent before participating in any study procedures. The trial was registered with ClinicalTrials.gov (NCT03009695).

Study design has been presented in detail elsewhere [11]; however, the total number of patients enrolled in the safety assessment is larger than the 33 patients enrolled in the first phase of the study by Ferrulli et al. [11].

Sixty-three patients with obesity, fulfilling all inclusion/exclusion criteria, were randomized to receive a 5-week treatment with rTMS: 31 were treated with HF rTMS (18 Hz; HF group), 13 were treated with LF rTMS (1 Hz; LF group), and 19 were sham treated (sham group). All patients underwent a total of 15 rTMS sessions (3 per week for 5 weeks).

The rTMS was performed by a trained physician using a Magstim Rapid^2^TMS (The Magstim Co. Ltd, Whitland, Carmarthenshire, UK) stimulator equipped with an H-shaped coil, which allows direct stimulation of deeper brain regions. Specifically, the deep rTMS has been addressed to bilaterally stimulate the prefrontal cortex (PFC) and the insula.

HF sessions consisted of 80 trains of 18 Hz, each lasting 2 s, with an intertrain interval of 20 s. The HF treatment duration was 29.3 min with 2880 pulses in total. LF sessions consisted of 4 trains of 1 Hz, each lasting 10 min, with an intertrain interval of 1 min. The LF treatment duration was 43 min with 2400 pulses in total. The Sham treatment was performed by a sham coil located in the same case of the real coil, producing similar acoustic artefacts and scalp sensations, inducing only negligible electric fields in the brain. In all groups receiving the real treatment, the stimulation was performed with an intensity of 120% of the resting motor threshold (RMT).

Follow-up visits were planned 1 month, 6 months, and 1 year after the end of the treatment.

Analysis of AEs also involved the patients who discontinued the treatment during the first phase of the clinical trial and were excluded from the statistical analysis as per protocol [11], as well as those patients enrolled after the end of the first phase of the protocol.

Adverse event (AE)‑related outcome measures patient data were recorded via electronic forms by physicians during each of 15 session visits and at follow-up visits (occurring after 1 month, 6 months and 1 year from the last TMS session), including safety/tolerability issues with neurostimulation treatment, presence of comorbid conditions and use of concomitant medications.

According to the Food and Drug Administration (FDA) definition, in this study we considered to be an AE any untoward medical occurrence associated with the use of the medical device (rTMS) in humans, whether they were considered related to rTMS procedure or not.

AEs were classified by type of side effect, frequency of occurrence, duration, relationship to the experimental treatment (possibly/probably/not related), seriousness (serious/not serious), and severity (mild/moderate/severe).

AEs were considered as “possibly related to treatment” if they occurred within a reasonable time sequence following TMS session and were biologically plausible. Alternatively, the AE could be explained by concurrent disease or other drugs/chemicals.

AEs classified as “probably related to treatment” were those that occurred within a reasonable time sequence following TMS session, were biologically plausible, and were unlikely to occur as a result of concurrent disease or other drugs/chemicals.

A serious adverse event (SAE) was defined as any AE (experience) that resulted in any of the following outcomes: death, life-threatening experience, inpatient hospitalization, or prolongation of existing hospitalization (for >24 h), persistent or significant incapacity or substantial disruption of the ability to conduct normal life functions, congenital anomaly/birth defect, or requiring an intervention to prevent permanent impairment or damage.

As per protocol, all the SAEs related to the study and unexpected (i.e., not listed in the protocol as an expected occurrence) have been emailed to the Research Ethics Committee (REC) and to the Italian Ministry of Health using the specific safety report form. These were sent within 15 days of the Principal Investigator becoming aware of the event. Reports of SAEs in double-blind trial were unblinded.

### Statistical analysis

A descriptive analysis was conducted both in the total population and in the three treatment groups (HF, LF, and Sham). Age and level of education reported at the time of enrollment were used to define three age subgroups and three educational subgroups, respectively. The three age subgroups were as follows: (1) subjects between 20 and 30 years old, (2) subjects between 30 and 50 years old, (3) subjects >50 years of age. The three education subgroups were as follows: (1) primary education, (2) high school diploma, and (3) university degree.

Differences in age, body weight, and BMI between the three subgroups (HF, LF, and Sham) were evaluated using the one-way ANOVA test; the Binomial test was used to evaluate the gender differences within the groups and for individual comparisons between the group reporting AEs (AEs group) and the group not reporting AEs (No AEs group); the Chi-square test was used for individual comparisons of AEs within and between the three treatment groups.

All statistical analyses were conducted using GraphPad software. Graphs were created with GraphPad software. Significance level was set at *p* < 0.05.

## Results

### Baseline demographics characteristics

From January 2017 to January 2020, a total of 63 subjects undergoing a 5-week rTMS experimental treatment for obesity were evaluated for possible side effects (48 F, 15 M; mean age 48.3 ± 10.4 years; mean body weight 97.9 ± 14.8 kg; mean BMI 36.3 ± 4.4 kg/m^2^) (Table 1).

**Table 1:** Socio-demographic characteristics and anthropometric measures of the entire population of subjects with obesity enrolled in the study

	Total	HF	LF	Sham	*p* value
Patients, *n* (%)	63	31 (49.2%)	13 (20.6%)	19 (30.2%)	0.0183*
Gender					
Female, *n* (%)	48 (76.2%)	23 (74.2%)	10 (76.9%)	15 (78.9%)	0.0681
Male, *n* (%)	15 (23.8%)	8 (25.8%)	3 (29.1%)	4 (21.1%)	0.2466
Body weight (kg)					
Mean ± SD	97.9 ± 14.8	97.7 ± 16.4	98.6 ± 17.3	97.9 ± 10.4	0.9832
BMI (kg/m^2^)					
Mean ± SD	36.3 ± 4.4	35.8 ± 4.8	36.8 ± 5.3	36.6 ± 3.2	0.7361
Age (years)					
Mean ± SD	48.3 ± 10.4	46.9 ± 10.3	49 ± 11.2	49.9 ± 10.3	0.5885
Age (years)					
Median (Q1, Q3)	48 (42, 57)	48 (41, 55)	47 (44.5, 57.5)	52 (41, 58)	–
Age (years)					
Range (Min, Max)	46 (22, 68)	38 (26, 64)	43 (22, 65)	36 (32, 68)	–
Age					
20–30 years, *n* (%)	5 (7.9%)	4 (12.9%)	1 (7.6%)	0 (0.0%)	–
30–50 years, *n* (%)	28 (44.5%)	14 (45.2%)	6 (46.2%)	8 (42.1%)	0.1561
>50 years, *n* (%)	30 (47.6%)	13 (41.9%)	6 (46.2%)	11 (57.9%)	0.2725
Education					
PE, *n* (%)	12 (19.0%)	3 (9.7%)	6 (46.1%)	3 (15.8%)	0.4724
HS, *n* (%)	35 (55.6%)	18 (58.1%)	5 (38.5%)	12 (63.1%)	0.0266*
UD, *n* (%)	16 (25.4%)	10 (32.2%)	2 (15.4%)	4 (21.1%)	0.0388*

Data are expressed as mean ± SD, percentage (%) or median (Q1–Q3). Comparisons between the three subgroups of the treatment (HF, LF, and Sham) have been performed by ANOVA-one-way test for body weight, BMI, and age and by Chi-square test for gender, ranges of age, and ranges of education. The Chi-square test has not been applied in 20–30 years range of age due to the absence of cases in the Sham group

*HF* high frequency, *LF* low frequency, *SD* standard deviation, *BMI* body mass index, *PE* primary education, *HS* high school, *UD* university degree

*p < 0.05

Out of 63 enrolled subjects, 31 underwent HF stimulation (23 F, 8 M; mean age 46.9 ± 10.3 years; mean body weight 97.7 ± 16.4 kg; mean BMI 35.8 ± 4.8 kg/m^2^), 13 received LF stimulation (10 F, 3 M; mean age 49.0 ± 11.2 years; mean body weight 98.6 ± 17.3 kg; mean BMI 36.8 ± 5.3 kg/m^2^), and 19 were Sham-treated (15 F, 4 M; mean age 49.9 ± 10.3 years; mean body weight 97.9 ± 10.4 kg; mean BMI 36.6 ± 3.2 kg/m^2^).

Other socio-demographic characteristics of participants are reported in Table 1.

No significant differences between the three groups were found at baseline for age, body weight, and BMI (Table 1). A significant difference was found between the numbers of male and female subjects both in total population (*p* < 0.0001) and in the HF and Sham subgroups (*p* < 0.05) (females > males).

### Adverse events (AEs)

#### AEs in the total population

**Fig. 1:**
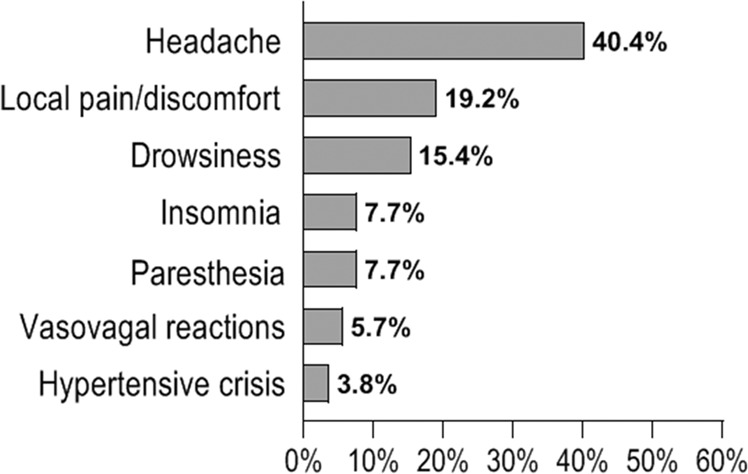
Distribution of all adverse events (AEs) reported by the total population of enrolled patients (*N*. 63 subjects with obesity). Data are expressed as percentage (%) of the total number of AEs (*N*. 52 AEs)

All AEs reported by the total population and their percentages are shown in Fig. 1.

Out of 63 enrolled patients, 32 subjects (50.8%) [22 F, 10 M (*p* = 0.0651); mean age 48.9 ± 9.9 years; mean body weight 99.3 ± 17.1 kg; mean BMI 36.5 ± 5.3 kg/m^2^] reported AEs (AEs Group), including mild, moderate, and severe events. The total number of reported AEs was 52. The comparison between severity levels of AEs showed a prevalence of mild and moderate intensity AEs over severe AEs [21 were of mild intensity (40.4%), 27 were moderate (51.9%), and 4 were severe (7.7%); *p* = 0.0003].

Out of 32 patients reporting AEs (AEs Group), 3 patients (9.4%) were 20–30 years old, 14 ranged between 30 and 50 years (43.8%), 15 patients (46.9%) were >50 years of age; about the level of education, 5 subjects (15.6%) attended primary school, 16 (50%) high school, 11 (34.4%) had a university degree.

Within the AEs group, no significant differences in age, BMI, and body weight were found between the subgroups (HF, LF, and Sham); the number of subjects who reported AEs was significantly higher in the HF group than in the other groups (*p* = 0.0020) (Table 2).

**Table 2:** Socio-demographic characteristics and anthropometric measures of the two subpopulations: subjects with obesity reporting AEs (AEs group) and not-reporting AEs (No AEs group)

AEs group						No AEs group				
	Total	HF	LF	Sham	*p* value	Total	HF	LF	Sham	*p* value
Patients, *n* (%)	32 (50.8%)	20 (62.5%)	7 (21.9%)	5 (15.6%)	0.0020*	31 (49.2%)	11 (35.5%)	6 (19.3%)	14 (45.2%)	0.2058
Gender										
Female, *n* (%)	22 (68.8%)	14 (70.0%)	5 (71.4%)	3 (60.0%)	0.0093*	26 (83.9%)	9 (81.8%)	5 (83.3%)	12 (85.7%)	0.2410
Male, *n* (%)	10 (31.3%)	6 (30.0%)	2 (28.6%)	2 (40.0%)	0.2019	5 (16.1%)	2 (18.2%)	1 (16.6%)	2 (14.3%)	0.8187
Body weight (kg)										
Mean ± SD	99.3 ± 17.1	101.7 ± 16.7	93.0 ± 20.7	98.6 ± 13.9	0.52	96.5 ± 12.1	90.3 ± 13.4	105.1 ± 10.3	97.6 ± 9.4	0.0427*
BMI (kg/m^2^)										
Mean ± SD	36.5 ± 5.3	36.5 ± 5.3	36.8 ± 7.1	36.3 ± 2.7	0.9877	36.0 ± 3.4	34.6 ± 3.7	36.8 ± 2.6	36.7 ± 3.4	0.2622
Age (years)										
Mean ± SD	48.9 ± 9.9	47.2 ± 10.6	50.7 ± 8.9	53 ± 8.8	0.4486	47.6 ± 11.0	46.4 ± 10.4	47.0 ± 14.1	48.9 ± 10.9	0.8538
Age (years)										
Median (Q1, Q3)	48 (45, 56.5)	48 (43, 55)	46 (45, 60)	57 (44.5, 59.5)	–	47 (41, 57)	47 (37, 54)	48.5 (38.5, 57.25)	49.5 (39.75, 57.75)	–
Age (years)										
Range (Min, Max)	39 (26, 65)	38 (26, 64)	24 (41, 65)	22 (39, 61)	–	46 (22, 68)	33 (27, 60)	42 (22, 64)	36 (32, 68)	–
Age										
20–30 years, *n* (%)	3 (9.4%)	3 (15.0%)	0 (0.0%)	0 (0.0%)	–	2 (6.4%)	1 (9.1%)	1 (16.6%)	0 (0.0%)	–
30–50 years, *n* (%)	14 (43.8%)	9 (45.0%)	4 (57.1%)	1 (20.0%)	–	14 (45.2%)	5 (45.4%)	2 (33.4%)	7 (50.0%)	0.2574
>50 years, *n* (%)	15 (46.9%)	8 (40.0%)	3 (42.9%)	4 (80.0%)	0.2466	15 (48.4%)	5 (45.4%)	3 (50.0%)	7 (50.0%)	0.4493
Education										
PE, *n* (%)	5 (15.6 %)	2 (10.0%)	2 (28.6%)	1 (20.0%)	–	7 (22.6%)	1 (9.1%)	4 (66.7%)	2 (14.3%)	–
HS, *n* (%)	16 (50 %)	11 (55.0%)	3 (42.8%)	2 (40.0%)	0.0104*	19 (61.3%)	7 (63.6%)	2 (33.4%)	10 (71.4%)	0.0759
UD, *n* (%)	11 (34.4%)	7 (35.0%)	2 (28.6%)	2 (40.0%)	0.1030	5 (16.1%)	3 (27.3%)	0 (0.0%)	2 (14.3%)	–

Data are expressed as mean ± SD, percentage (%) or median (Q1–Q3). Comparisons between the three subgroups of the treatment (HF, LF, and Sham) have been performed by ANOVA-one way test for body weight, BMI, and age and by Chi-square test for gender, ranges of age, and ranges of education. The Chi-square test has not been applied in 20–30 years range of age due to the absence of cases in the Sham group

*HF* high frequency, *LF* low frequency, *SD* standard deviation, *BMI* body mass index, *PE* primary education, *HS* high school, *UD* university degree

*p < 0.05

Comparing the three subgroups (HF, LF, and Sham) within the AEs group, the number of female subjects was significantly higher in HF compared with the other two subgroups (*p* = 0.0093).

Socio-demographic and anthropometric characteristics of No AEs Group are shown in Table 2. A prevalence of female subjects has also been observed in this group (*p* = 0.0003).

Comparing the AEs group with the No AEs group, no significant differences were found in the number, gender, age, education, body weight, and BMI (*p* > 0.05), both in the total group and in the subgroups (HF, LF, and Sham), except for a higher number of female subjects in the Sham subgroup of the AEs group compared with the Sham subgroup of No AEs group (*p* = 0.0352).

#### AEs in subgroups HF, LH, and Sham

**Fig. 2:**
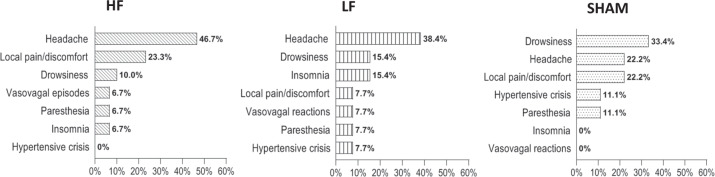
Distribution of the reported adverse events (AEs) in the two groups of the treatment: high frequency (HF) and low frequency (LF), and in the Sham group. Data are expressed as percentage (%) of the total AEs in the HF (*N*. 30, 57.7%), LF (*N*. 13, 25.0%), and Sham (*N*. 9, 17.3%) groups

Distribution of AEs in the two arms of the treatment (HF and LF) and in Sham group is shown in Fig. 2.

Out of 52 reported AEs, 30 occurred in HF (57.7%), 13 in LF (25%), 9 in Sham (17.3%). The difference in the number of AEs was statistically significant between the three treatment subgroups (*p* = 0.0008), with a prevalence in the HF (Table 3).

**Table 3:** Complete list of adverse events (AEs) and their severity levels in the AEs Groups and in the 3 treatment subgroups

	AEs group	HF	LF	SHAM	*p* value
Total AEs	52	30 (57.7%)	13 (25.0%)	9 (17.3%)	0.0008**
Mild	21 (40.4%)	11 (36.7%)	5 (38.5%)	5 (55.6%)	0.1801
Moderate	27 (51.9 %)	16 (53.3%)	7 (53.8%)	4 (44.4%)	0.0060**
Severe	4 (7.7%)	3 (10.0%)	1 (7.7%)	0 (0.0%)	–
Headache	21 (40.4%)	14 (66.7%)	5 (23.8%)	2 (9.5%)	0.0038**
Mild	6 (24.6 %)	2 (14.3%)	2 (40.0%)	2 (100%)	–
Moderate	13 (61.9%)	10 (71.4%)	3 (60.0%)	0 (0.0%)	–
Severe	2 (9.5%)	2 (14.3%)	0 (0.0%)	0 (0.0%)	–
Local pain/discomfort	10 (19.2%)	7 (70.0%)	1 (10.0%)	2 (20.0%)	0.0450*
Mild	4 (40.0%)	3 (42.8%)	0 (0.0%)	1 (50.0%)	–
Moderate	6 (60.0%)	4 (57.1%)	1 (100.0%)	1 (50.0%)	–
Severe	0 (0.0%)	0 (0.0%)	0 (0.0)	0 (0.0%)	–
Drowsiness	8 (15.4%)	3 (37.5%)	2 (25.0%)	3 (37.5%)	0.08825
Mild	6 (75.0%)	2 (66.6%)	2 (100.0%)	2 (66.6%)	–
Moderate	2 (25.0%)	1 (33.3%)	0 (0.0%)	1 (33.3%)	–
Severe	0 (0.0%)	0 (0.0%)	0 (0.0%)	0 (0.0%)	–
Insomnia	4 (7.7%)	2 (50.0%)	2 (50.0%)	0 (0.0%)	–
Mild	2 (50.0%)	2 (100%)	0 (0.0%)	0 (0.0%)	–
Moderate	2 (50.0%)	0 (0.0%)	2 (100%)	0 (0.0%)	–
Severe	0 (0.0%)	0 (0.0%)	0 (0.0%)	0 (0.0%)	–
Paresthesia	4 (7.7%)	2 (50.0%)	1 (25.0%)	1 (25.0%)	–
Mild	2 (50.0%)	1 (50.0%)	1 (100.0%)	0 (0.0%)	–
Moderate	2 (50.0%)	1 (50.0%)	0 (0.0%)	1 (100.0%)	–
Severe	0 (0.0%)	0 (0.0%)	0 (0.0%)	0 (0.0%)	–
Vasovagal reactions	3 (5.7%)	2 (66.7%)	1 (33.3%)	0 (0.0%)	–
Mild	1 (33.3%)	1 (50.0%)	0 (0.0%)	0 (0.0%)	–
Moderate	1 (33.3%)	0 (50.0%)	1 (100.0%)	0 (0.0%)	–
Severe	1 (33.3%)	1 (50.0%)	0 (0.0%)	0 (0.0%)	–
Hypertensive crisis	2 (3.8%)	0 (0.0%)	1 (50.0%)	1 (50.0%)	–
Mild	0 (0.0%)	0 (0.0%)	0 (0.0%)	0 (0.0%)	–
Moderate	1 (50.0%)	0 (0.0%)	0 (0.0%)	1 (100%)	–
Severe	1 (50.0%)	0 (0.0%)	1 (100%)	0 (0.0%)	–

Data are expressed as percentage (%) of the total number of AEs (N.52) both in AEs group and in the subgroups (HF, LF, and Sham). The percentages of a single AE refer to the total AEs of each subgroup (HF, LF, and Sham). The percentages relative to the severity levels of AEs refer to the number of each single AE. Comparisons between the three subgroups of the treatment (HF, LF, and Sham) have been performed by Chi-square test for each analyzed variable. The Chi-square test has been applied only to those comparisons where the number of groups allowed it

*AE* adverse events, *HF* high frequency, *LF* low frequency

*p < 0.05; **p < 0.001

Within HF, 11 AEs (36.7%) were of mild, 16 (53.3%) of moderate, 3 of severe intensity (10%) (*p* = 0.0136); within LF, 5 AEs (38.5%) were mild, 7 (53.8%) moderate, 1 severe (7.7%) (*p* = 0.116); within Sham, 5 AEs (55.6%) were mild, 4 (44.4%) moderate, 0 severe.

Out of total 52 AEs, the most frequently reported side effect was headache (N. 21, 40.4%); headache intensity was mild in 6 cases (24.6%), moderate in 13 (61.9%), severe in 2 (9.5%) (*p* = 0.019) (Table 3).

Out of 21 total headache-related episodes, 14 cases occurred in HF (66.7%), 5 in LF (23.8%), and 2 in Sham (9.5%), with a significant size difference between the three groups (*p* = 0.0038). Headache was mainly of moderate intensity both in HF (71.4%) and in LF (60%); headache in the Sham group occurred only with mild intensity. The number of headache events of mild intensity was the same in the three groups (No 2).

The mean duration of headaches was shorter in the in HF compared to other groups [2.2 ± 0.8 (HF) vs 3.1 ± 0.1 (LF) vs 2.8 ± 0.5 (Sham) h; *p* = 0.0456]. In the HF, most headache episodes (71.4%) occurred within the first 5 TMS sessions, 21.4% occurred between the 1st and the 10th TMS session, only one episode (7.2%) between the 10th and the 15th TMS session reflecting the percentages of the total group.

Out of 14 patients reporting headache in HF, 5 (35.7%) used medication to relieve the symptom; three patients out of 5 in the LF group used medication (60%). No patient resorted to medication in the Sham group.

The second most frequently reported side effect was local pain/discomfort (19.2%); it occurred in 10 patients (15.9%); intensity of local pain/discomfort was mild in 4 (40%), moderate in 6 (60%), severe in 0 cases. Under no circumstances this side effect was severe. Out of 10 total local pain/discomfort events, 7 cases occurred in HF (70%), 1 in LF (10%), and 2 in Sham (20%), with a significant size difference between the 3 subgroups and a prevalence in HF (*p* = 0.0450) (Table 3).

In HF, local pain/discomfort was of mild (42.8%) or moderate (57.1%) intensity; in LF, the only case reported was of moderate intensity; in Sham, 1 case was mild and 1 case was of moderate intensity.

The mean duration of local pain/discomfort was 1.6 ± 0.8 h, without significant differences between the three subgroups (HF, LF, and Sham). Local pain/discomfort occurred mainly within the first 5 TMS sessions (70%); in the HF, most episodes (85.7%) occurred within the first 5 TMS sessions, 14.3% occurred between the 1st and 10th dTMS session, no episode occurred after the 10^th^ TMS session.

Out of 7 patients in HF, only 2 (28.6%) used medication to relieve local pain/discomfort; in LH, the only patient reporting this AE used medication; in the Sham, no patient used specific medication.

Concerning the other side effects (drowsiness, insomnia, paresthesia, vasovagal reactions, hypertensive crisis), no significant differences in numbers were found within both groups and subgroups. In the Sham group, drowsiness was the most frequently reported AE (33.4%).

#### Dropped-out patients

Eight patients (12.7%, 8 F), out of the 63 enrolled patients, dropped out from the study.

Four subjects (50%) decided to voluntarily stop treatment for personal reasons (HF group, *n* = 2; Sham group, *n* = 1; LF group, *n* = 1). One patient (HF group, *n* = 1) decided to withdraw her consent from the experimental study following a vasovagal reaction. The event occurred during the fourth session of HF TMS, after ~5 pulses. The participant was sitting on a chair with her back and thighs supported and both feet on the floor, she started feeling dizzy and faint, without losing consciousness and experiencing seizures. The patient appeared pale and sweating, blood pressure was 80/50 mmHg and hearth rate was 92 bpm. The participant was moved to a lying down position. During the 20-min period following the episode, the patient stated that she felt tired, dizzy, anxious, and nauseated. The patient continued to be monitored by medical staff until the symptoms completely disappeared, ~120 min after the event.

Hypertensive episodes were the cause of dropout in another patient (LF group, *n* = 1), who already had a history of high blood pressure and was taking antihypertensive drugs. Hypertensive episodes started after the third TMS session and were associated with headache. Due to a poor response to therapy and the persistence of high blood pressure episodes, after the sixth TMS session, the investigators decided to stop treatment, with the patient’s consent.

Two additional patients discontinued the study for incidental reasons not related to the experimental treatment: one patient (LF) reported traumatic right shoulder bone fracture, the other patient reported an asymptomatic incidental meningioma (HF) diagnosed while performing a head MRI. The study was discontinued to allow the patients to proceed with the necessary therapeutic itinerary.

#### Serious adverse events (SAEs)

According to the definition reported in the “Materials and method” section, the two previously reported AEs (traumatic right shoulder bone fracture, and asymptomatic incidental meningioma) were considered SAEs, together with a single case of left unilateral hearing loss, associated with dizziness. This event occurred 1 month after the end of the treatment in a 60 year old patient suffering from diabetes, hypercholesterolemia and hypertension. The patient was enrolled in the LF group, and used ear plugs during the entire duration of the treatment as per protocol. The patient reported the AE by phone and refused to come to our center for a check-up visit; she did not provide any clinical documentation relative to this AE. Therefore, a close correlation with the rTMS treatment cannot be demonstrated.

All three SAEs were reported to the REC and to the Ministry of Health using the specific safety report form, within 15 days of the Principal Investigator becoming aware of the event.

## Discussion

To our knowledge, this is the first analysis that compares safety and tolerability of deep rTMS in subjects with obesity, treated with either high frequency, low frequency, or sham stimulation.

It is well known that individuals with obesity exhibit an altered sensory detection and pain sensitivity, and consequently, a higher risk of developing side effects from neurostimulation. The interaction of genetic, metabolic, biomechanical, environmental, behavioral, social, and cultural factors seems to be involved in the increased susceptibility to pain in obesity [26]. For example, the generation of pro-inflammatory cytokines by adipose tissue could result in sensitization of nociceptors and central nociceptive transmission pathways [27]. Genetic mutations of signal molecules produced by the adipose cell (e.g. leptin) appear also to be involved in the individual responses to physiologic, environmental, and psychological stresses seen in both obesity and painful syndrome [28]. Furthermore, dysfunction of dopaminergic, serotoninergic, endocannabinoid systems in the reward circuitry underlies an array of behavioral problems in obesity, such overeating, pain catastrophizing, kinesiophobia, and depression, leading probably to emphasize side effects [29].

Findings from our analysis did not reveal any new or unexpected safety concerns, and in relation to the already known side effects of rTMS, only headache and local pain/discomfort have been significantly more frequent in the HF group, compared to LF and Sham. No significant differences were found in the occurrence of other AEs clusters among the two analyzed treatment groups and the Sham group. Furthermore, in this study we verified the safety and tolerability of rTMS up to 1 year from the end of the treatment, supporting the good long-term tolerability of this treatment, previously not extensively investigated in other TMS safety studies.

Headache was the most frequently reported side effect (33.3% in total population; 66.7% in HF, 23.8% in LF and 9.5% in the Sham group). It was mainly moderate in intensity, lasted about 3 h, and disappeared in the majority of patients within the first five sessions of deep rTMS treatment. It has long been known that headache is the most common TMS-associated side effect [1]. The percentage of headache occurrence varies among the different clinical trials, ranging from 11% [30] up to 65% [14, 31]. The features of this AE vary according to scalp location of the coil (i.e., headache as well as neck pain appear to be more frequent when rTMS has been applied outside the motor area) [14], coil design, intensity [i.e., use of supraliminal intensities (>100% of the RMT)] [32], frequency of stimulation, and individual susceptibility [1]. The stimulation protocol, used in our clinical trial, providing for repetitive modality, supraliminal intensity of stimulation (120% of RMT), PFC area location, could account for a high percentage of subjects who reported headache, especially in HF group. In our study, no patient experienced headache for the whole duration of the study, and this side effect disappeared within a maximum of 5 days from the beginning of the treatment, moving towards tolerance. In apparent contradiction, several studies support the evidence that rTMS may be a beneficial treatment option for patients with headache and migraine [33]. The mechanisms underlying migraine encompass neural and vascular causes, including cerebral cell hyperexcitability, sensitization of the trigeminovascular pathway, genetics, and environmental factors [34].

Single pulse and rTMS proved to be a promising non-pharmacological intervention for headache and migraine [35]. This effect is obtained by stimulating the primary motor cortex (M1), consequently inhibiting the activity of the thalamus and therefore pain perception, and activating the dorsolateral prefrontal cortex (DLPFC), leading to a decreased activity of the midbrain-medial thalamic pathway related to pain relief.

The increased β endorphin levels induced via rTMS was hypothesized as a possible mechanism involved in headache relief in patients with migraine, especially if rTMS was applied at HF and addressed to DLPFC. This is because patients with migraine usually present lower plasma β endorphin levels [36]. The U.S. FDA cleared TMS as a conventional treatment for migraine. In line with previous reports on migraine patients, we demonstrated that HF deep rTMS treatment, directed to the PFC, determines an increase of β-endorphin level in obese subjects [37]. This finding suggests a possible role of β-endorphins in early extinction and in moderate intensity of headache side effect. The greater susceptibility to migraine, that people with obesity show, should also be taken into consideration [38].

TMS not only generates electrical currents in brain tissue, but stimulates excitable superficial tissue, including scalp muscles and peripheral nerves, provoking strong contractions of scalp, head, and mostly neck muscles [39, 40]. Inferior frontal and temporal locations of stimulation are associated with more considerable discomfort, compared to superior and posterior scalp locations. Accordingly, in our clinical trial in which stimulation is addressed to the PFC bilaterally, local pain and discomfort represented the most frequently reported side effect following headache. Paresthesia (which occurred only in few cases in our study), together with itching and burning are among the most commonly reported rTMS side effects in adults [41].

Magnetic pulses cause mechanical vibrations in the coil, producing a brief but very loud sound (coil click) that may exceed 140 dB of sound pressure level, exceeding the recommended safety levels for the auditory system [42]. Therefore, the sound pulse can potentially cause hearing loss, although this risk can be counteracted with adequate hearing protection. However, cases of transient increases in auditory thresholds, and a single case of permanent threshold shift in a single individual who did not wear ear plugs have been reported [1]. In our study, about a month after the end of the experimental treatment, one patient receiving LF stimulation, developed a left unilateral hearing loss, associated with dizziness symptoms.

Although hearing loss is recognized to be a side effect of TMS treatment, it is unlikely that in this case the hearing loss was associated with the TMS treatment for several reasons: the long time elapsed (about 1 month) between the end of the treatment and the onset of symptoms, the presence of risk factors for other pathologies that may have led to hearing loss (e.g., sensorineural hearing loss induced by ischemic injury in cochlear microcirculation), the use of adequate hearing protection, and the low frequency of the treatment received. It has been shown that the amplitude of rTMS noise is directly linked to the coil design, the absolute stimulation intensity, which is tailored to each subject’s RMT, and the frequency of stimulation, with the greatest energy at high frequencies (from 2 to 7 kHz) [43].

Given the intrinsic neural connections between brain and heart, an influence of brain stimulation techniques, such as rTMS and transcranial direct current stimulation, on cardiovascular system functioning is conceivable. In our study, we reported two cases of hypertensive crisis (1 in LF and 1 in Sham) and three cases of vasovagal reactions (2 in HF and 1 in LF).

The sympathetic activation control of the cardiovascular system involves many structures at different levels of central (CNS) and peripheral (PNS) autonomic nervous system. Two different levels of regulation can be distinguished: a “bottom-up regulation” in which feedbacks from PNS activity, circulating hormones, and secretion of neuropeptides by the adenohypophysis are integrated and processed by the nuclei of the brainstem; and a “top-down regulation”, in which several cortical brain areas (e.g., sensorimotor cortex, the medial PFC and the insular cortex) modulate PNS activity, and consequently, influence the cardiovascular system [44]. Several studies hypothesized that TMS could affect the autonomic nervous control of the cardiovascular system through the stimulation of the above-mentioned brain areas. An Italian study reported that LF rTMS of the PFC induces a slight parasympathetic activation (highlighted by a significant bradycardia), and no changes in the sympathetic function [45]. Conversely, HF rTMS producing cortical excitation especially when applied to the primary motor cortex has been supposed to evoke cardiac responses mediated by connections in the brain cortex with the cardiac-related centers of the CNS (e.g., increase in heart rate) [46]. In our study, contrary to expectations, neither significant changes in blood pressure nor hypertensive crisis were observed in obese patients receiving HF stimulation. In reviewing previous studies, there is no consensus on rTMS effects on sympathetic system [47–51]. Effects of rTMS on the autonomic function should therefore be investigated with specifically designed studies. It is not presently possible to establish a causal link between the two episodes of hypertensive crisis, occurred in the LF and Sham groups, with the treatment.

In contrast, the evidence of rTMS effects on the parasympathetic system are more solid. A study showed that 12 sessions of HF rTMS addressed to the left PFC induced a significant reduction in the sympathetic/parasympathetic ratio, suggesting an improvement of vagal activity [52]. In this study, 3 vasovagal reactions occurred in HF (*n*. 2) and LF (*n*. 1) groups, indicating a possible modulatory action by the rTMS on the parasympathetic system. However, the number of events is so negligible that it is difficult to establish whether these episodes were secondary to an emotional response (triggered by anxiety, noxious stimuli, prolonged standing) or direct effect of TMS on autonomic nervous system function. Exclusion of any history of syncopal events prior to undergoing the rTMS procedure is mandatory to ensure the safety of patients.

In this study, rTMS has been specifically addressed to the PFC and insula, bilaterally. The insular cortex is integrated in the neural system which is involved in the processing of external sensory information, and is responsible for the neural control of appetite and the regulation of energy balance [53]. The PFC plays a role in executive and cognitive functions, including inhibitory control, and an impaired activation of PFC has been reported in individuals with obesity. High frequency rTMS over the PFC alters cortical excitability through the modulation of different neurotransmitters, in brief, inducing dopamine release and enhancing GABA neurotransmission, with consequent increased cortical inhibitory activity [54]. This mechanism has been hypothesized to underlie the deep rTMS capacity in controlling the food craving and then, inducing weight loss in obesity [11], but it does not seem to be directly involved in the pathogenesis of side effects. Quite the opposite, concerning the headache, the left PFC stimulation might exert an inhibitory effect on pain perception by activation of supra-spinal pathways or by resetting the fronto-limbic dysfunction, or by increasing the basal low plasma β-endorphin levels, observed in chronic painful conditions [55]. The exact cause of TMS-related headache is not entirely clear, it is thought to be caused by the activation of muscles and nerves near the stimulation coil, which results in contraction/twitches of the scalp and upper face muscles in some patients [30]. About the seizures, the most severe acute adverse effect of rTMS, they are not strictly linked to the stimulation of the cortex PFC, but are caused by hypersynchronized discharges of groups of neurons in the gray matter, mainly due to an imbalance between inhibitory (e.g., GABA) and excitatory (e.g., dopamine, glutamate) synaptic activity in favor of the latter [1]. The risk of developing seizures significantly increases when safety guidelines related to stimulation protocol, inclusion/exclusion criteria, individual motor threshold determination, are not observed.

This study has some limitations that must be considered when interpreting the results. First, the sample is small and potentially unrepresentative of the large number of individuals with obesity treated with rTMS (also for other neuropsychiatric disorders). Furthermore, in this study the last follow-up visit has been performed after 1 year from the last rTMS session. If, on the one hand, this allows us to verify the possible onset of late side effects, on the other hand the self-reported side effects are hardly attributable to the experimental treatment or to other causes, due to the long time since the last stimulation. Finally, the great variability of the stimulation protocols and the large number of neuropsychiatric disorders treated with TMS explain the wide rate variability of occurrence of some side effects in rTMS clinical trials (e.g., headache and discomfort).

In conclusion, our study confirmed the safety profile of rTMS in the long term and for the first time in the population of subjects with obesity. Notwithstanding the fact that individuals with obesity exhibit an altered sensory detection and pain sensitivity, a higher incidence of the most common TMS-associated AEs was not elicited in comparison with previous literature in obesity.

Moreover, our trial did not reveal any new or unexpected safety concerns. Nevertheless, the collection of a detailed medical history is strongly recommended to exclude possible risk factors for AEs and SAEs, when applying rTMS to obese subjects.

## Data Availability

Data that support the findings of this study are available from the authors, upon request.

## Abbreviations

rTMS: repetitive transcranial magnetic stimulation
AEs: adverse events
BMI: body mass index
HF: high frequency
LF: low frequency
RMT: resting motor threshold
FDA: Food and Drug Administration
SAE: serious adverse event
REC: Research Ethics Committee
MRI: magnetic resonance imaging
DLPFC: dorsolateral prefrontal cortex
PFC: prefrontal cortex
dB: decibel
CNS: central nervous system
PNS: peripheral nervous system
